# Differential diagnosis of sensory modulation disorder (SMD) and attention deficit hyperactivity disorder (ADHD): participation, sensation, and attention

**DOI:** 10.3389/fnhum.2013.00862

**Published:** 2013-12-16

**Authors:** Aviva Yochman, Osnat Alon-Beery, Ahuva Sribman, Shula Parush

**Affiliations:** ^1^School of Occupational Therapy, Faculty of Medicine of Hadassah and the Hebrew University of JerusalemJerusalem, Israel; ^2^Child Developmental Center, Meuhedet Healthcare OrganizationJerusalem, Israel

**Keywords:** sensory modulation, attention deficit hyperactivity disorder, sensory processing, attention, participation, differential diagnosis

## Abstract

Differential diagnosis between sensory modulation disorder (SMD) and attention deficit hyperactivity disorder (ADHD) is often challenging, since these disorders occur at a high rate of co-morbidity and share several clinical characteristics. Preliminary studies providing evidence that these are distinct disorders have focused solely on body functions, using sophisticated laboratory measurements. Moreover, no studies have compared participation profiles of these populations. This study is the first to compare the profiles of these populations regarding both “body functions” (attention and sensation) and “participation,” using measures applicable for clinical use. The study included 19 children with ADHD without SMD and 19 with SMD without ADHD (diagnosed by both pediatric neurologists and occupational therapists), aged 6–9, and matched by age and gender. All children underwent a broad battery of evaluations: the Evaluation of Sensory Processing, Fabric Prickliness Test (FPT) and Von Frey Test to evaluate sensory processing, and Test of Everyday Attention to evaluate attention components. The Participation in Childhood Occupations Questionnaire was used to evaluate participation. Results support significant group differences in all sensory components, including pain intensity to suprathreshold stimuli and pain “after sensation,” as well as in tactile, vestibular, taste, and olfactory processing. No differences were found in attention components and participation. This study has both theoretical and clinical importance, inter alia, providing further evidence of two distinct disorders as well as indications of specific clinical instruments that might enable clinicians to implement differential diagnoses. In addition, results accord with other previous statements, which indicate that the clinical diagnosis of children with disabilities may not be a major factor in determining their participation profile.

## Introduction

Attention deficit hyperactivity disorder (ADHD) is one of the most prevalent and intensively studied childhood developmental disorders (Barkley, 2003). It is characterized by a persistent pattern of inattention, and/or hyperactivity- impulsivity, to a degree that causes significant impairment of functional performance at home, school, and in social settings (American Psychological Association [APA], 2013). Estimated prevalence rates of ADHD vary greatly (Froehlich et al., 2007); however, the results of population surveys suggest that in most cultures ADHD occurs in about 5% of children (APA, 2013).

The literature indicates that ADHD is often accompanied by deficits other than those subsumed under the ADHD diagnosis. In fact, the subject of co-occurring deficits is one of the most frequently explored aspects of this disorder (Adesman, 2003; Gillberg et al., 2004). Findings of both clinical and community studies have revealed extremely elevated rates of co-occurrence between ADHD and other neuro-developmental disorders, predominantly related to motor (e.g., Pitcher et al., 2003), language (e.g., Cohen et al., 2000), cognitive (e.g., Frazier et al., 2004) and sensory functioning (e.g., Yochman et al., 2006). Pertaining to the sensory domain, children with ADHD frequently meet the criteria for sensory modulation disorder (SMD) as well (Miller et al., 2001).

SMD is characterized by difficulty in responding to sensory input in a graded and adaptive manner relative to the degree, nature, or intensity of the sensory input. Furthermore, individuals with SMD routinely respond to benign sensory input with exaggerated avoidant and defensive behaviors that are inappropriate to the environmental demands (Miller et al., 2007). These behaviors range from over to under- responsiveness to sensory stimuli and/or intensely seeking sensory stimuli, and may involve only one or multiple sensory systems (Dunn, 1997; Miller et al., 2007). Studies have shown that individuals with SMD present with behavioral and physiological features of sensory processing that are different from those of typically developing children (McIntosh et al., 1999a; Reynolds and Lane, 2008; Bar-Shalita et al., 2009a,b; Davies et al., 2010). For sensory processing difficulties to be classified as a disorder, an individual's responses to sensory input must significantly impair his/her successful performance of daily activities and routines (Bar-Shalita et al., 2008). The prevalence of SMD is estimated to be 5–16% in the normal population (Ahn et al., 2004; Ben-Sasson et al., 2009; Gouze et al., 2009), similar to that of ADHD.

Differential diagnosis between SMD and ADHD is often challenging, since these disorders share several clinical characteristics. The behavioral responses of children with sensory over-responsivity in the face of adverse sensory stimulation may manifest as distractibility, impulsivity, hyperactivity, or some combination of these, which represent the core symptoms of ADHD (Miller et al., 2012). In addition to sharing behavioral characteristics, several studies have revealed a high prevalence of co morbidity—over half the children with ADHD may also exhibit SMD (Mangeot et al., 2001; Miller et al., 2001; Yochman et al., 2006)—increasing the difficulty of the differential diagnosis process. Researchers have employed both behavioral and physiological measures in an attempt to describe the unique sensory responsivity patterns of children with ADHD compared to those exhibited by typically developing children. Results of behavioral measures such as parent questionnaires, have indicated that children with ADHD are more sensitive to sensory stimuli, such as tactile, visual, auditory and oral, than typical children (Dunn and Bennet, 2002; Yochman et al., 2004). Studies that employed physiological measures, such as the central Somatosensory Evoked Potential (SEP) (Parush et al., 1997), and sympathetic markers of nervous system functioning using electro-dermal reactivity (EDR), (McIntosh et al., 1999a; Mangeot et al., 2001; Miller et al., 2001) have also indicated that the responses of a significant percentage of children with ADHD differ from those of typical children, suggesting stronger physiological reactivity.

Despite the similarity between children with SMD and those with ADHD with respect to these and other clinical characteristics, preliminary studies comparing the two populations have provided evidence that these disorders are indeed separate, each with its own unique profile. Thus, for example, results of a study that compared children with ADHD and tactile over-responsivity to children with ADHD without tactile over-responsivity, demonstrated significantly higher SEP amplitudes in the group with sensory modulation difficulties (Parush et al., 2007). In addition, the preliminary research of Lane et al. (2010) led them to suggest that patterns of salivary cortisol and electrodermal responsivity to sensation may distinguish between groups of children with ADHD with and without sensory over-responsivity. More recently, the study of Miller et al. (2012) revealed that children with SMD have larger EDR responses to sensory stimuli and exhibit more somatic complaints, anxiety and depression than children with ADHD.

The current study is comparative, examining differences between children with a sole diagnosis of ADHD to children with a sole diagnosis of SMD in an attempt to determine whether these disorders are distinct. While there are a very few studies that have compared such groups of children, their focus is mainly on body functions utilizing sophisticated equipment. In addition, to our knowledge, no other study has compared the participation profiles of these children across multiple areas of functioning. The World Health Organization (WHO) posits that participation is directly related to health and represents the highest level of functioning (WHO, 2001). Although only limited research has been performed with respect to the participation profiles among children with ADHD and/or children with SMD, the evidence to date suggests that the participation of these children is significantly impaired in various aspects of daily life compared to typically developing children (Cohn et al., 2000; Harpin, 2005; Dunn, 2007; Bar-Shalita et al., 2008; Engel-Yeger and Ziv-On, 2011). A comparison between these two diagnostic populations regarding the unique expression of their participation limitations may prove to be an additional important factor in their differential diagnosis.

The uniqueness of this study lies in it being the first to compare the profiles of ADHD and SMD regarding both “body functions” (sensation and attention) and “participation,” through the use of clinically applicable measures. A better understanding of the specific features that distinguish between these two disorders can enable a more accurate differential diagnosis process, and may have a profound impact on intervention planning.

## Materials and methods

### Participants

Participants in the study were recruited from a major developmental center in Israel. Of the 63 children referred for the study, 15 were excluded due to their having a dual diagnosis of both ADHD and SMD. Ten other children could not be included because their parents chose to withhold their consent. Thus, the final sample was composed of 38 children; 19 children with ADHD without SMD (11 male, 8 female; mean age 6 years and 8 months [*SD* = 7 months]; age range 6–8.11 years) and 19 with SMD without ADHD (13 male, 6 female; mean age 6 years and 7 months [*SD* = 8 months]; age range 6–8.4 years). No group differences were found with respect to age [*t*_(36)_ = 0.630; *p* = 0.533] and gender [χ^2^(1) = 452; *p* = 0.501].

The ADHD group included children who scored as such on the CPRS-R:S (Connors, 1997) and as typically behaving on the Short Sensory Profile (McIntosh et al., 1999b). The opposite was true for the children included in the SMD group. Children in the SMD group scored as having definite deficits on the Short Sensory Profile and as typically behaving on the CPRS-R:S. To further verify the presence or absence of ADHD according to the DSM-IV criteria, as well as to exclude children with additional physical and/ or neurological deficits (e.g., cerebral palsy, ASD), all children underwent an additional evaluation by a developmental neurologist. Moreover, participants were evaluated by an occupational therapist to substantiate the presence or absence of SMD.

### Procedure

Following research approval and parental consent, children were recruited for the study according to the inclusion criteria. Prior to receiving therapeutic or medical intervention, each child was individually evaluated on a broad battery of evaluations by an established occupational therapist with 10 years of experience working with this population. In addition, mothers completed the relevant questionnaires. The examiner was blind as to group placement.

### Instrumentation

#### Baseline measures

***The short sensory profile (SSP; McIntosh et al., 1999b)***. A standardized parent-report questionnaire used to screen children between the ages of 3–10 for sensory modulation deficits as well as for research purposes. The questionnaire contains 38 items reflecting responsiveness to sensory input across sensory modalities, including tactile, auditory, visual, gustatory, olfactory stimuli, movement, and body position. Parents indicate their perception of the frequency with which their child exhibits atypical behavioral responses to sensory stimulation on a 5-point Likert scale ranging from 1 (always) to 5 (never). Higher scores represent more functional performance. A total score was calculated for each participant by summing the item scores. Construct validity of the SSP has been demonstrated using the “known-groups” procedure and factor analysis. Convergent validity was established through electrodermal response testing, which has shown that abnormal electrodermal responses are significantly associated with lower scores on the SSP. Cronbach's alpha coefficient values ranged from 0.70 to 0.90, demonstrating internal consistency reliability (McIntosh et al., 1999a). The Hebrew version of SSP was found to have good psychometric properties (Engel-Yeger, 2010).

***Conners' Parent Rating Scale- Revised: Short Form (CRPS-R:S; Connors, 1997)***. The CRPS-R:S is a parent-report tool for 3–17 year old children to assess behaviors associated with ADHD according to the criteria referred to in the DSM-IV-TR (APA, 2000). Items also relate to various behaviors that may accompany attention disorders reflecting anxiety, conduct, and emotional problems. The CRPS-R: S includes 27 items grouped into four subscales: oppositional, Hyperactivity, Cognitive Problems/Inattention, and ADHD index. Each item is rated on a 4-point Likert scale ranging from 0 (not true at all/never) to 3 (very much true/very often) indicating the occurrence of the behavior over the previous month. Item scores are summed individually for each subscale and total subscale scores are compared to the standardized scores. The authors report medium—high internal consistency reliability (Cronbach's alpha 0.85 to 0.93) and test-retest reliability from 0.62–0.85, *p* < 0.05 for all the scales. The tool significantly discriminates between ADHD and non-ADHD populations (*p* < 0.001) and high criterion validity was reported (Kumar and Steer, 2003).

### Study measures

#### Sensory measures

Both the Fabric Prickliness Test (FPT) and the von Frey Monofilament Test used in this study (see below) are based on quantitative sensory testing (QST); a psychophysical approach used to characterize somatosensory hypersensitivity in a non-invasive but rigorous manner. Participants rate the subjectively perceived intensity of controlled graded levels of stimuli (Verdugo and Ochoa, 1992; Hansson et al., 2007; Arendt-Nielsen and Yarnitsky, 2009). Both tests have been shown to be valid methods of determining pain levels in children, as well as for measuring and comparing pain and pain “after sensation” between children with SMD and typically developing children (Bar-Shalita et al., 2009a,b).

***The Fabric Prickliness Test.*** (FPT; Garnsworthy et al., 1988; Cervero et al., 1994). This test quantifies the level of pain evoked by the application of prickly fabrics to the skin. In the present study, 16 applications of three types of woolen fabrics with different levels of prickliness (least prickly, mildly prickly, and very prickly) were used for each child. The different fabrics were applied face down (to prevent visual identification) to the volar surface of the child's non-dominant forearm and presented sequentially in an identical pseudorandom order for each child. Using digits 2–4, the investigator rapidly tapped on each individual fabric, repeating this sequence until the child verbally indicated registering the sensation. At this point, the fabric was removed and the child was asked to rate the level of pain the fabric evoked using the Revised Faces Pain Scale (FPS-R; Hicks et al., 2001). This scale presents schematic drawings of six faces expressing increasing levels of distress typically experienced by individuals with pain. The faces correspond to a numerical rating scale ranging from 0 to 10 with increments of 2, with the higher score representing the highest level of pain. Pain “after-sensation” (the duration in which the sensation of pain continues to linger) was measured after the last fabric was scored by having the child indicate when he/she no longer feels the sensation. The first “after sensation” measurement was taken 15 s after the final FPT fabric was scored and measurements were then repeated at 1-min intervals thereafter. The time taken for the sensation to dissipate was recorded.

***Pinprick pain (Smith and Nephew Rolyan; Menomonee Falls, WI)***. A series of Von-Frey filaments were used to test pinprick pain. Three stiff mono-filaments with variable bending forces were applied perpendicularly to the skin on the volar surface of the child's dominant forearm. Each filament was applied three times, for a total of 9 applications. The filaments elicit increasing levels of punctate pain by applying a bending force of 5.46, 5.88, and 6.10 on a log force scale (29 g, 75 g, and 127 g; 284.4 mN, 735.5 mN, 1245.4 mN, respectively). The filaments were applied in an identical pseudorandom order to each child. Children wore a blindfold during each application to prevent visual cues of the stimuli, which was then removed to rate pain intensity. Pain intensity was then rated using the FPS-R (Hicks et al., 2001) as detailed above.

***The Evaluation of Sensory Processing Questionnaire***. (ESP; Parham and Johnson-Ecker, 2002). The ESP is a standardized behavioral care-giver questionnaire designed to identify behaviors that are specifically indicative of sensory processing problems in 5–12 year old children. The ESP is the predecessor of the Sensory Processing Measure Home questionnaire (SPM) (Kuhaneck et al., 2007). The ESP provides scores of function in the visual, auditory, tactile, olfactory/gustatory, proprioceptive, and vestibular sensory systems. It is distinctive in that it contains only items that are specific to particular sensory systems (Parham and Johnson-Ecker, 2002). Each item is rated according to the frequency of the behavior using a 5-point Likert scale. The standard score for each of the subscales enables the classification of children's functioning into one of three interpretive ranges: typical performance, probable dysfunction or definite dysfunction. Studies on the psychometric properties of the ESP revealed Cronbach's alphas of 0.83 or above on most scales (Johnson-Ecker and Parham, 2000) and inter-rater reliability; when examining mother- father agreement in their responses about their child, parent agreement was found across more than 75% of the items (Chang, 1999). LaCroix and Mailloux (1995) conducted a validity study in which parents were asked to rate their typically developing preschoolers according to the ESP items. On the majority of items, 75% or more of the parents responded that the items describe behaviors that are not typical of preschoolers. Criterion validity using contrasting groups showed that many of the items were rated significantly higher by children with autism (Vermass Lee, 1999) and children with sensory processing deficits (Johnson-Ecker and Parham, 2000) than by typically developing children.

#### Attention measure

***The test of everyday attention for children***. (TEA-Ch; Manly et al., 1999). The TEA-Ch is a standardized measure designed to assess various components of attention in children aged 6–16. The test comprises nine “game-like” subtests that require visual, auditory, and motor skills to measure the child's ability to selectively attend, sustain attention, divide attention, switch attention and inhibit verbal and motor responses. The developers selected assessment tasks designed to minimize potential confounding factors such as motor speed, reading and writing and memory, so that the targeted attentional system be activated (Heaton et al., 2001). The current study employed the five subtests (Sky Search, Score, Creature Count, Sky Search Dual Task and Walk, Don't Walk) recommended by the developers to be used for screening purposes. The following is a description of the five subtests administered:

1. Sky Search—Examines selective visual attention by measuring the speed and accuracy with which one scans a test sheet with numerous visual stimuli to select identical pairs of stimuli (“spaceships”) from the unpaired distractor stimuli, while controlling for motor efficiency.
2. Score—Assesses sustained auditory attention. The participant silently counts the number of target tones, which are presented at varying intervals.
3. Creature counting—Examines attentional switching and control. The children are asked to repeatedly switch between forward and backward counting of visual stimuli aligned along a path in response to arrows pointing upward and downward. The target stimuli are located within an array of visual stimuli.
4. Sky Search Dual Task—Assesses sustained and divided attention, indicating the ability to perform two tasks simultaneously. Respondents must identify identical pairs of visual stimuli from visual distractors (as in Sky Search), while simultaneously counting tones presented at fixed intervals.
5. Walk, don't walk—Assesses sustained attention and response inhibition. Respondents progress along a paper path (marking steps with a pen) in response to a “go” sound, but are to refrain from doing so when hearing a “no-go” tone.

The TEA-Ch was standardized on 293 Australian children (Manly et al., 1999). Construct validity was established through factor analysis (Manly et al., 1999; Passantino, 2011). In addition, the criterion validity of various TEA-Ch subtests was examined by comparing them to other measures of attention. Passantino (2011) found statistically significant correlations between the Sky Search (*r* = 0.40, *p* < 0.001) and Map Mission (*r* = 0.31, *p* < 0.01) tasks and the Stroop measure; and between the Sky Search (*r* = 0.69, *p* < 0.001) and Map Mission (*r* = 0.37, *p* < 0.001) tasks and the Trails Test A. Studies have also found that children with ADHD performed significantly worse than typically developing children on the subtests assessing sustained attention and attentional control/switching, but not on the subtest of selective attention (Heaton et al., 2001; Manly et al., 2001). Test-retest reliability was assessed on a random subgroup of 55 children across age groups from the original sample and correlation coefficients ranging from 0.64 to 0.92 were obtained (Manly et al., 2001).

#### Participation measure

***The participation in childhood occupations questionnaire (PICO-Q; Bar-Shalita et al., 2009a,b)***. This is a standardized reliable and valid caregiver questionnaire validated on an Israeli population of children. This questionnaire was designed to evaluate participation in four areas of functional activities: personal activities of daily living; academic activities; play and leisure, and habits and routines. Each item describes an activity that is scored according to three different scales: (1) level of activity performance, (2) level of enjoyment of the activity, and (3) frequency of performance of the activity. Each of these scales provides scores for each of the four individual performance areas. A total score is also calculated for each individual scale. In addition, this questionnaire provides descriptive data by having parents select one of nine characteristics or behavior patterns that they feel underlie their child's participation difficulties. Reliability has been established through internal consistency (Cronbach's *a* = 0.86–0.89) and test–retest (*r* = 0.69–0.86) measures. Content and construct validity have been demonstrated (Bar-Shalita et al., 2009a,b).

### Data analysis

Data were analyzed using both parametric and non-parametric statistics, depending on the variable's distribution. Multivariate analysis of variance (MANOVA) was used to analyze group differences in scores obtained on the ESP, the TEA-Ch and the PICO-Q. The von Frey and FPT did not meet criteria for normal distribution (Komogorov–Smirnov <0.05), therefore group comparisons were performed through the Mann–Whitney test.

In addition to the comparisons performed between the two diagnostic populations of this study, a comparison of the PICO-Q scores between children with ADHD and typically developing children, as well as between children with SMD and typically developing children was performed using one sample t-tests. This comparison was possible in view of the fact that data regarding the functioning of typically developing Israeli children on the PICO was reported by Bar-Shalita et al. (2008).

## Results

### Differences between groups on sensory measures:

Results support significant group differences on all sensory measures, which indicate significantly greater sensory difficulties in the SMD group.

### Pinprick pain test

Significant differences were found between the groups on the overall Von-Frey filament test score (*Z* = −2.24; *p* = 0.026). The children with SMD reported higher scores as a response to punctate pain (median = 60) compared to children with ADHD (median = 30).

#### The fabric prickliness test

Significant differences were found between the groups in the level of pain elicited by the application of the fabrics (*Z* = −2.367; *p* = 0.018), such that children with SMD reported higher scores (median = 16) compared to children with ADHD (median = 4). In addition, significant group differences were found in the measures of pain “after-sensation” (*Z* = −2.803; *p* = 0.005). After the application of the last fabric of the FPT, the after pain sensation in children with SMD lingered longer (median = 2 min, 15 s) than the children with ADHD (median = 15 s).

#### The evaluation of sensory processing questionnaire (ESP)

The results of the MANOVA on the six subtest scores revealed a significant group effect [*F*_(1, 35)_ = 5.950; *p* < 0.001]. To examine the source of the effect, a univariate analysis of variance (ANOVA) was conducted for each of the individual subtests. The results indicate that the scores for the SMD group were significantly lower than the scores of the ADHD group in three of the six subtests (i.e., taste and smell, tactile and motion /vestibular) (Table 1).

**Table 1:** **Results of multivariate analyses of variance (MANOVA) comparing test scores on the ESP between children with ADHD and children with SMD**.

**Subsection**	**Children with ADHD (*n* = 19)**		**Children with SMD (*n* = 19)**		***F***	***P***	**Effect size (partial eta squared)**
	**M**	***SD***	**M**	***SD***			
Hearing	41.42	6.736	38.53	6.834	1.73	0.197	0.046
Taste and smell	22.26	2.621	18.58	3.702	12.53	0.001	0.258
Body awareness	50.95	14.547	44.21	7.458	3.23	0.081	0.082
Touch	92.11	28.276	69.32	10.878	10.75	0.002	0.230
Motion (vestibular)	60.47	6.222	53.63	8.565	7.94	0.008	0.181
Vision	45.95	8.263	49.42	11.177	1.19	0.283	0.032

ESP, Evaluation of Sensory Processing; ADHD, attention deficit hyperactivity disorder; SMD, sensory modulation disorder.

### Differences between groups on the attention measure

A comparison between groups using a MANOVA analysis revealed no significant group differences on any of the TEA-Ch subtests [*F*_(1, 27)_ = 0.655, *P* = 0.686], indicating that the children with ADHD did not perform worse than the children with SMD on the various attention sub-tests.

### Differences between groups in the participation measure

#### Quantitative data

To determine group differences on the PICO- Q scores, a MANOVA was performed on the total scores of the three questionnaire scales (performance level, degree of enjoyment of activity and frequency of performance), as well as on the participation scores obtained in each performance area (daily care activities, academic activities, play and leisure and habits and routines). Only in the “degree of enjoyment” for “daily care activities” [*F*_(1, 36)_ = 5.97; *p* = 0.020] did the results reveal any significant difference between the groups, indicating that children with SMD enjoy these activities less (*M* = 34.58; *SD* = 9.73) than children with ADHD (*M* = 41.05; *SD* = 6.21).

Table 2 displays the results of a one sample *t*-test used to compare the participation of each of the experimental groups (ADHD, SMD) to typically developing children. Data regarding the typical sample was based on the information reported by Bar-Shalita et al. (2009a,b). Significant differences were found between children with SMD and typically developing children [*t*_(18)_ = 6.011, *p* = 0.000]; as well as between children with ADHD and typically developing children [*t*_(18)_ = 3.72, *p* = 0.001] on the total “level of performance” dimension of participation, indicating that both experimental groups obtained mean scores significantly below those reported for typically developing children. In contrast, no differences were found between the experimental groups compared to typically developing children on the dimensions of “enjoyment” and “frequency of performance.”

**Table 2:** **Results of PICO-Q scores for the three dimensions of participation, according to study groups**.

**Dimension of participation**	**Children with ADHD (*n* = 19)**		**Children with SMD (*n* = 19)**		**Typical children (*n* = 34)**	
	**M**	***SD***	**M**	***SD***	**M**	***SD***
Level of activity performance	127.95	22.92	121.79	17.88	148.53	10.04
Enjoyment of activity	127.58	19.65	118.11	21.19	127.18	12.11
Frequency of performance	75.63	17.28	70.42	4.55	67.60	12.05

PICO-Q, Participation in Childhood Occupations Questionnaire; ADHD, attention deficit hyperactivity disorder; SMD, sensory modulation disorder.

#### Descriptive data

Different trends were found between the ADHD and SMD groups with respect to the responses of parents regarding their perception of the reasons underlying their children's participation difficulties. Thus, for example, a higher percentage of children with ADHD reportedly had difficulty due to poor quality of performance or the length of time they required to perform activities. In contrast, a higher percentage of parents reported that their children with SMD had difficulty due to inflexibility, fighting with their parents or refusing to participate (Table 3).

**Table 3:** **PICO-Q: Comparison of behavior characteristics of children with ADHD and SMD underlying poor performance as reported by parents**.

**Behavior characteristics (as reported by parents)**	**Children with ADHD (***n*** = **19**)**	**Children with SMD (***n*** = **19**)**
	% reported	%reported
Poor quality of performance	26.98	11.74
Performance time longer than expected	26.98	13.73
Completes task only with constant arguing /bribing/ lack of flexibility	17.10	35.79
Refuses to perform task	7.82	27.45
Does not follow appropriate rules of behavior	15.85	8.34
Performs the task too often	0.00	0.00
Does not perform task enough	5.27	2.95

PICO-Q, Participation in Childhood Occupations Questionnaire; ADHD, attention deficit hyperactivity disorder; SMD, sensory modulation disorder.

## Discussion

Children with ADHD demonstrate significant functional performance impairments at home, school, and in social settings (APA, 2013). In addition to the impairment caused by the core symptoms of ADHD, these children are at increased risk of associated deficits in various areas, including sensory processing (Mangeot et al., 2001; Miller et al., 2001). Thus, sensory processing ability is one of the many factors that need to be considered when assessing the reasons why a child with ADHD may be experiencing difficulties participating in daily activities. However, consideration of these issues in the evaluation and treatment of children with ADHD is often challenging due to the significant overlap of ADHD and SMD symptoms (Miller et al., 2001; Ahn et al., 2004; Gouze et al., 2009).

An important question raised in recent studies is whether ADHD and SMD are distinct disorders, the same disorder or co-morbid disorders (Miller et al., 2012). To date, very few studies compared children who only meet the criteria for one or the other diagnosis—children with SMD without ADHD, and ADHD without SMD—so that the unique characteristics of each can be used to discriminate between the two conditions. Therefore, the purpose of this study was to compare children with a sole diagnosis of SMD and a sole diagnosis of ADHD on the central underlying symptoms of both disorders. Furthermore, due to the fact that participation is becoming increasingly important in the field of childhood disability, this study compared the participation profiles of children with SMD to those with ADHD in all areas of functioning.

### Differences between groups according to sensory measures

The results of this study demonstrated significant group differences on all sensory measures, indicating that the children in the SMD group had significantly greater sensory difficulties than those in the ADHD group.

Specifically, the parent-report measure (ESP) revealed significant group differences in several sensory systems (tactile, gustatory/olfactory and movement/vestibular). These findings are in line with the study performed by Miller et al. (2012), in which the SSP was used to compare four groups of children: children with SMD, with ADHD, with SMD + ADHD and typically developing children. Supporting the results of the current study, Miller et al. found that children with SMD obtained significantly poorer scores than children with ADHD in the areas relating to tactile, taste/smell, and movement sensitivity. However, they also found significant differences in visual-auditory sensitivity. It is important to note that Miller and colleagues found significant differences in these sensory domains between children with SMD and typically developing children, but not between children with ADHD and typically developing children. This supports the suggestion that the behavioral manifestations of these sensory systems may be more characteristic of children with SMD than children with ADHD and hence, may be useful in their differential diagnosis.

Although it is difficult to demonstrate the distinction between SMD and ADHD through behavioral analysis, data derived from parent report questionnaires are often an important component in the clinical diagnoses of both SMD and ADHD (Johnson-Ecker and Parham, 2000; Tripp et al., 2006; Reynolds and Lane, 2008). However, in addition to behaviors indicative of sensory processing *per se*, some sensory questionnaires also address clinically significant problem behaviors considered to be derivatives of sensory processing deficits—such as those related to attention and social-emotional functioning (Yochman et al., 2004), which can also be found among children with a broad range of disabilities including ADHD (Koziol and Budding, 2012). In contrast, the ESP was uniquely designed to identify behaviors that are indicative specifically of sensory processing problems in the various sensory systems (Johnson-Ecker and Parham, 2000). Given the clinical and theoretical importance of determining the characteristics that can distinguish between SMD and ADHD, researchers, and clinicians should consider using sensory processing evaluation tools with a higher level of specificity than those used to differentiate between SMD and typically developing children alone.

In addition to the ESP, psychophysical performance-based evaluations that are practical and appropriate for clinical use were employed. Previous research has shown that children diagnosed with SMD reported higher levels of pain than those reported by typically developing children in response to both pinprick (Von Frey monofilaments) and prickly fabrics, suggesting that children with SMD demonstrate a more vigilant nociceptive system. In addition, pain “after-sensation” to prickly fabrics was found to linger for at least 5 min after the termination of the test among children with SMD (Bar-Shalita et al., 2009a,b).

The present study is the first to compare between children with SMD and ADHD using these psychophysical methods. Results of our study indicate that children with SMD reported significantly higher levels of pain than those reported by children with ADHD on both pinprick (von Frey monofilaments) and prickly fabrics. Moreover, the children with SMD reported feeling pain for a significantly longer time than the children with ADHD, indicating increased “after-sensation” to the stimuli. These results support the findings of Bar-Shalita et al. (2009a,b), suggesting that one of the definitive features of children with SMD is increased aversive responses to suprathreshold tactile stimuli—which reflects abnormal central processing of nociceptive input—as compared to typically developing children. The results of the present study add to the previous results by demonstrating that such responses are not typical of children with ADHD, suggesting that children with ADHD do not have abnormalities in processing suprathreshold noxious tactile sensations (Arendt-Nielsen and Yarnitsky, 2009).

Our finding that children with SMD experience significantly longer pain “after sensation” compared to children with ADHD is also noteworthy. Clinical parent reports regarding children with SMD often describe that their children feel aversive sensations long after the sensory stimuli has been terminated (i.e., feeling pain long after a child got hit, or continuing to display aversive smell responses from an object long after it has been removed). Our results regarding “after sensation” seem to be in accord with the limited research done on the habituation profiles of these populations. The few studies that assessed sympathetic “flight or fight” reactions of children with SMD in response to sensory stimuli as measured by electrodermal activity (EDA) found that these children exhibited exaggerated electrodermal responses to sensory stimulation, and habituate more slowly to repeated stimulation than do typically developing children (McIntosh et al., 1999a; Miller et al., 2001, 2012). However, the physiological reactivity profile of children with ADHD has been shown to be different from that of children with SMD. Variability exists with regard to the magnitude of their response to stimuli (Miller et al., 2012), being either smaller (Mangeot et al., 2001) or the same (Herpertz et al., 2001) as typically developing children, and a faster than normal habituation to repeated stimulation has been demonstrated (Mangeot et al., 2001; Miller et al., 2001). These results, together with the results of the present study, seem to suggest that there are differences between these populations regarding the ability of these children to habituate sensory stimuli.

In summary, the findings of group differences on these sensory measures provide additional supporting evidence that SMD is a separate clinical condition distinct from ADHD. With further research in larger samples, the clinical tests used in this study may prove to be useful for differential diagnosis.

### Differences between groups on the attention measure

Although attention difficulties have been found to be characteristic of children with SMD (Dunn, 1997; Miller et al., 2012), as well as in children with ADHD, the assumption is that this difficulty is not a core symptom of the disorder as it is in ADHD, but rather a secondary behavioral manifestation of the sensory over-responsivity experienced by children with SMD to adverse sensory stimulation. Therefore, we hypothesized that children with SMD would perform better than children with ADHD on the measure of attention used. In contrast to our assumption, the MANOVA analysis revealed no significant group differences on any of the five TEA-Ch subtests administered (Sky Search, Score!, Creature Counting, Sky Search Dual Task, Walk Don't Walk).

Studies performed to attempt to discriminate between SMD and ADHD have mainly focused on areas related to the core symptoms of SMD. Only one other study, to our knowledge, compared these populations with regard to measures of attention. Unlike the findings of the present study, Miller et al. (2012) found that although both children with ADHD and children with SMD had significantly more attention problems compared to typically developing children, children with ADHD had significantly worse attention scores than children with SMD. These results were found on both the Parent Leiter international performance scale as well as on the and SNAP- IV parent rating scale for the assessment of ADHD. Nevertheless, no group differences were found on the Child Behavior Checklist (CBCL), a parent report scales which assesses a variety of behaviors, including attention problems. Miller et al. (2012) note that it is common to find differing results when measuring similar constructs with different tools.

Thus, a possible explanation for our results may be related to the instrument chosen for this study. The TEA-Ch was used to measure attention in this study because of its reported advantages. That is, it was found to be a reliable performance-based attention measure (as opposed to parent-report questionnaires) that relates to multiple components of attention, it is ecological valid, and is unique due to the game-like nature of the tasks. Nevertheless, according to the test developers the subtests of the TEA-Ch do not purport to measure attention directly. Rather, they measure differences in performance abilities believed to contribute significantly to inferred separable attention processes, including auditory and visual detection, counting ability, processing speed, and response speed among other factors (Manly et al., 1999). In addition, the studies utilizing the TEA-Ch with ADHD study samples are not always consistent with respect to their results regarding which attention components are impaired among children with ADHD compared to typically developing children (Manly et al., 1999, 2001; Heaton et al., 2001; Villella et al., 2001; Lajoie et al., 2005). This inconsistency has also been found in a number of other studies, using a variety of attentional measures (Wu et al., 2002; Berlin et al., 2003; Koschack et al., 2003).

The unsolved issue with regard to the classification and characterization of attentional components (Sergeant, 1996; Knudsen, 2007) as well as which attentional components are in fact impaired among children with ADHD (Wilding, 2005; Sutcliffe et al., 2006; Knudsen, 2007), causes further complications when trying to differentiate between developmental disabilities such as SMD and ADHD. There is also a question regarding the representativeness of our study sample. Specifically, the relative proportions of ADHD subgroups were not controlled for.

Future studies need to use more sensitive measures both in performance- based as well as behavioral inventories, which may shed light on the cognitive differences between SMD and ADHD. Furthermore, it is important to relate to additional defining characteristics of ADHD, such as deficits in executive functions (Barkley, 2003; Wilding, 2005), which have not yet been sufficiently examined with respect to their presence or absence in SMD.

### Differences between the groups on the participation measure

In comparing the quantitative data obtained through the PICO-Q between children with SMD and ADHD, the only significant difference between the groups was that the children with SMD had a lesser “degree of enjoyment” for “daily care activities.”

This is understandable, given that children with sensory over-responsivity experience these activities as unpleasant or threatening and not enjoyable. Children with over-responsivity are often characterized by behaviors such as limited preference for types of food, avoidance of various clothing materials, and/or dislike washing due to the feeling of running water or the smell of soap (Miller and Fuller, 2006; Reynolds and Lane, 2008).

Our findings indicate that, with the above exception, children with ADHD and children with SMD exhibit similar characteristics of participation in all three domains (level of activity performance, level of enjoyment and frequency of performance) and across multiple areas of function (activities of daily living, academic activities, play and leisure, habits, and routines).

A unique feature of this study is in comparing comprehensive participation profiles of children with ADHD and SMD across life situations. The majority of studies performed regarding the characteristics of participation among children with SMD and ADHD, have compared children with disabilities to typically developing children. On the whole, the results of these studies point to the fact that these children are at risk for limited participation in many aspects of daily life. This was also found to be the case in the present study, in which parents of both experimental groups rated the level of their child's participation abilities in activities throughout the day to be significantly poorer than those reported for typically developing children; a finding supporting those of previous studies both on SMD (Cohn et al., 2000; Dunn, 2001; Bundy et al., 2007; Bar-Shalita et al., 2008) as well as on ADHD (Cermak, 2005; Harpin, 2005).

By comparison, only a limited amount of studies have compared between different diagnostic populations in general, in order to identify the unique expression of participation limitations characteristic of different disability populations. Supporting the findings of the current study, these comparison studies seem to indicate a lack of significant group differences between clinical populations on participation measures. This is in accord with current perspectives on participation and health, indicating that the clinical diagnosis of children with disabilities is not a major factor in determining their participation profiles. Rather, meaningful participation in a given activity appears to depend on a variety of contextual and personal factors (King et al., 2003; Rosenberg et al., 2012).

Thus, for example, Law et al. (2004) examined the relationship between diagnosis, function, and participation among 427 children with physical disabilities. The sample was divided into one of two diagnostic categories—central nervous system-related disorders and musculoskeletal disorders. Using the Children's Assessment of Participation and Enjoyment (CAPE; King et al., 2004) the researchers revealed that when adjusted for age, gender and physical function, no significant differences were found in the participants' intensity, and diversity of participation over and above the level of function. Similar findings were also reported by Eriksson (2006) from a series of studies that included children with a variety of impairments (e.g., social skills, communication limitations, behavioral problems, low general health, visual impairments, physical impairments, and multiple impairments). She concluded that intensity and diversity of participation seems to be more related to personal and environmental factors than to disability type. Thus, further research should investigate the contribution of other confounding personal and environmental factors on the participation of children with disabilities in general, and when comparing between children with ADHD and SMD in particular.

It is interesting to note that the descriptive findings did find different trends between the groups with respect to parent's perceptions regarding the reasons underlying their children's participation difficulties. In fact, qualitative research on the unique expression of participation limitations of children with neurodevelopmental disorders is extremely limited. Due to the vast influence of participation on the development of competence, emotional well-being, and quality of life of a child (Law, 2002; Rosenberg et al., 2012), further studies should additionally explore the qualitative aspects of participation, which may provide a more in depth and informative approach to the study of the complex construct of participation.

## Conclusions

The present study is, to our knowledge, the first to compare the profiles of ADHD and SMD regarding the core symptoms of each of these disorders as well as their participation profiles. In addition, the instrumentation selected was comprised solely of practical and clinically applicable measures. Certain limitations of the research need to be taken into account when relating to the findings. This study included a small convenience sample of children, inter alia due to the difficulty of identifying participants with only one of these diagnoses. In addition, controlling for subtypes of ADHD and SMD was not performed. Furthermore, although all children attended mainstream public schools, the cognitive abilities of these children were not directly evaluated and may have influenced their performance. A further possible limitation is that, although all the tools have adequate psychometric properties, some have not been specifically validated for the local population.

Taking these limitations into account, our findings provide a number of important contributions to the existing literature, with the aim of providing a more comprehensive and in-depth understanding of the relationship between these deficits. Given the high risk of comorbidity in children with ADHD, the American Academy of Pediatrics (APA, 2000) recommend that clinicians routinely and systematically screen for comorbidity over and above the behavioral symptoms of ADHD, which may have motivated the initial referral (Adesman, 2003). The clinical implications derived from the results of this study support the practice of considering co-occurring sensory processing abilities among children with ADHD and may contribute to the process of differential diagnosis. Improved diagnostic accuracy is essential to providing a child with the most appropriate treatment.

### Conflict of interest statement

The authors declare that the research was conducted in the absence of any commercial or financial relationships that could be construed as a potential conflict of interest.
